# miR-29b defines the pro-/anti-proliferative effects of S100A7 in breast cancer

**DOI:** 10.1186/s12943-014-0275-z

**Published:** 2015-01-27

**Authors:** Helong Zhao, Tasha Wilkie, Yadwinder Deol, Amita Sneh, Akaansha Ganju, Mustafa Basree, Mohd W Nasser, Ramesh K Ganju

**Affiliations:** Department of Pathology, The Ohio State University Wexner Medical Center, 840 BRT, 460W 12th Ave, Columbus, OH 43210 USA

**Keywords:** Breast cancer, Cell proliferation, S100A7, miR-29b, p53

## Abstract

**Introduction:**

S100A7 (Psoriasin) is an inflammatory protein known to be upregulated in breast cancer. However, the role of S100A7 in breast cancer has been elusive, since both pro- and anti-proliferative roles have been reported in different types of breast cancer cells and animal models. To date, the mechanism by which S100A7 differentially regulates breast cancer cell proliferation is still not clear.

**Methods:**

We used Gene Functional Enrichment Analysis to search for the determining factor of S100A7 differential regulation. We confirmed the factor and elaborated its regulating mechanism using *in vitro* cell culture. We further verified the findings using xenografts of human breast cancer cells in nude mice.

**Results:**

In the present study, we show that S100A7 significantly downregulates the expression of miR-29b in Estrogen Receptor (ER)-positive breast cancer cells (represented by MCF7), and significantly upregulates miR-29b in ER-negative cells (represented by MDA-MB-231). The differential regulation of miR-29b by S100A7 in ER-positive and ER-negative breast cancer is supported by the gene expression analysis of TCGA invasive breast cancer dataset. miR-29b transcription is inhibited by NF-κB, and NF-κB activation is differentially regulated by S100A7 in ER-positive and ER-negative breast cancer cells. This further leads to differential regulation of PI3K p85α and CDC42 expression, p53 activation and p53-associated anti-proliferative pathways. Reversing the S100A7-caused changes of miR-29b expression by transfecting exogenous miR-29b or miR-29b-Decoy can inhibit the effects of S100A7 on *in vitro* cell proliferation and tumor growth in nude mice.

**Conclusions:**

The distinct modulations of the NF-κB – miR-29b – p53 pathway make S100A7 an oncogene in ER-negative and a cancer-suppressing gene in ER-positive breast cancer cells, with miR-29b being the determining regulatory factor.

**Electronic supplementary material:**

The online version of this article (doi:10.1186/s12943-014-0275-z) contains supplementary material, which is available to authorized users.

## Introduction

The inflammatory protein S100A7 (Psoriasin) was discovered as a marker of human psoriasis lesion [1,2]. S100A7 is mainly expressed in epithelial cells [2], and can be induced by pathogens and inflammatory cytokines [3,4]. It has been noticed that S100A7 is upregulated in breast cancer cells [5-7]. However, the role of S100A7 in breast cancer progression has been elusive, since both pro- and anti-proliferative roles have been reported in different types of breast cancer cells and animal models [8-11]. It has been shown that S100A7 promotes cancer growth and metastasis in basal-like (ER^−^) breast cancer cells [10,12]. Accordingly, S100A7 upregulation correlates with poor prognosis for patients with basal subtype breast carcinoma [9]. Conversely, it inhibits cancer growth and cell migration in luminal (ER^+^) breast cancer cells [8]. To date, the mechanism by which S100A7 differentially regulates ER^−^ and ER^+^ breast cancer cell proliferation is unknown.

microRNAs bind to untranslated regions (UTRs) of mRNAs, which inhibits protein translation and/or degrades mRNAs [13]. miR-29b is considered to be a tumor suppressor in multiple types of cancers [14,15], including breast cancer [16,17]. The anti-cancer effect of miR-29b has been shown to be related to its targeting of the 3′ UTRs of multiple key cancer regulators, thus suppressing the growth and metastasis of breast cancer. miR-29b is encoded by two genes, mir-29b-1 on chromosome 7 and mir-29b-2 on chromosome 1. It has been shown that NF-κB binds to the promoter of mir-29b-1 and inhibits its transcription [18]. In addition, NF-κB has been shown to transactivate YY1, which then binds to the promoter of mir-29b-2 and inhibits its transcription [19]. Since NF-κB governs numerous survival genes and apoptotic genes, its functions in cancer development has long been noted. The activity of NF-κB can be either upregulated or downregulated in cancer cells, depending on whether it is inducing survival signaling or apoptotic signaling [20]. The differential activation statuses of NF-κB in different breast cancer subtypes has led to the discovery of reciprocal regulation of NF-κB by ER [21].

Among the targets of miR-29b, PI3K p85α and CDC42 have been shown to regulate p53 activation [22]. Activated p53 translocates into the nucleus and activates multiple anti-proliferative pathways, including the ones of DNA repair and mitosis check point [23]. It was shown that miR-29b targets p85α and CDC42 and consequently inhibits p53 activation and cell proliferation [22].

In the present study, we described a novel role of the NF-κB – miR-29b – p53 pathway, which defines the distinct effects of S100A7 on regulating cell proliferation and tumor growth of ER^−^ and ER^+^ breast cancer.

## Results

### S100A7 differentially regulates proliferation of ER^−^ and ER^+^ breast cancer cells

To investigate the effect of S100A7 upregulation in human breast cancer, we generated a panel of S100A7 overexpressing breast cancer cell lines [8,10]. S100A7 overexpression significantly increased the proliferation of ER^−^ MDA-MB-231 cells and decreased that of ER^+^ MCF7 cells (Table 1). Furthermore, with S100A7 overexpression, MDA-MB-231 cells gained mesenchymal properties (fibroblast-like cell shape and absence of cell-cell adhesion) and increased tumor growth in nude mice; in contrast, with S100A7 overexpression, MCF7 cells gained epithelial properties (flat scale-like cell shape and increased cell-cell adhesion) and showed reduced tumor growth in nude mice (Table 1). These observations are in accordance with previous publications from our group and others [8,10,12]. In this study, we used MDA-MB-231 and MCF7 as representative cellular models of ER^−^ and ER^+^ breast cancers to analyze the mechanism by which S100A7 may differentially regulate cell proliferation.

**Table 1:** **Differential effects of S100A7 overexpression in human breast cancer cell lines**

**Cell line**	**MDA-MB-231**	**MCF7**
Cancer subtype	Basal-like	Luminal
ER status	ER negative	ER positive
Proliferation change with S100A7 overexpression	↑ 57%*	↓ 33%*
Morphology change with S100A7 overexpression	Gained mesenchymal	Gained epithelial
Tumor growth with S100A7 overexpression	↑ 1089%*	↓ 79%*

Changes of cell proliferation, morphology, tumor formation in nude mice caused by S100A7 overexpression in given cell lines are listed. Mesenchymal properties of cell morphology include fibroblast-like cell shape, absence of cell-cell adhesion; epithelial properties include flat and scale-like cell shape, increased cell-cell adhesion and clustering. (*p < 0.05).

### S100A7 overexpression induced differential miR-29b expression changes in MDA-MB-231 and MCF7 cells

**Figure 1:**
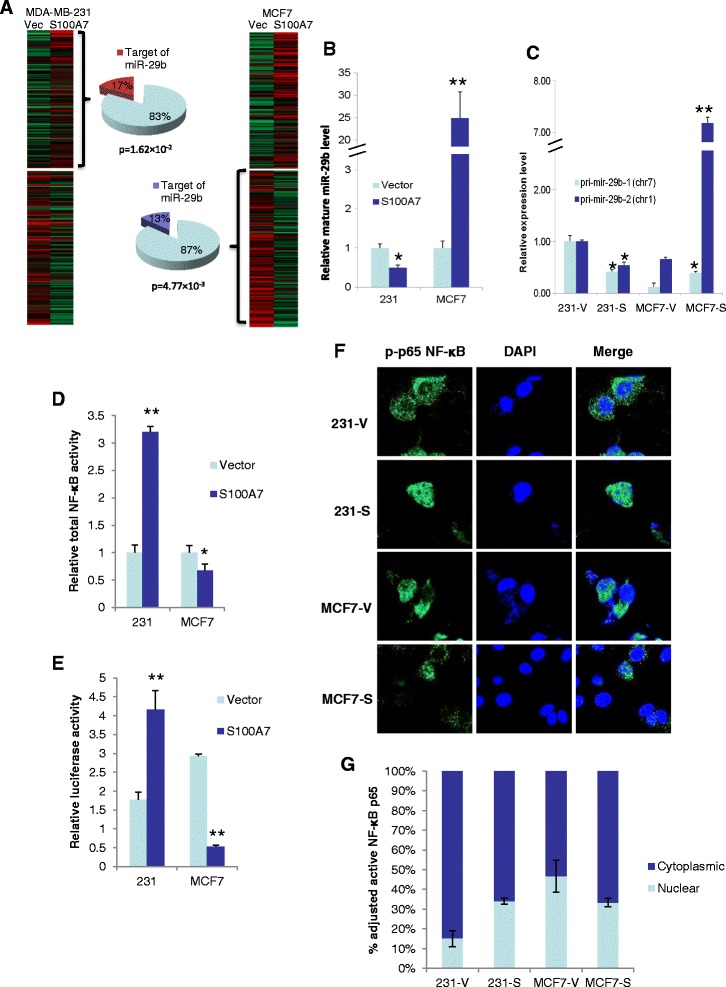
**S100A7 differentially regulates miR-29b transcription and NF-κB activation in MDA-MB-231 and MCF7 cells. (A)** GFE analysis was performed on the top up/down-regulated genes of 231-S100A7 and MCF7-S100A7 cells. Genes up/down-regulated > 2 folds in 231-S100A7 and those up/down-regulated > 2.5 folds (similar amounts of genes) in MCF7-S100A7 were filtered from microarray data and used as training sets for separate analysis. Training sets from microarray data are indicated by heat maps, and percentage of miR-29b targets are indicated with pie charts with significance of enrichment analysis. **(B)** mature miR-29b was measured by qRT-PCR in the given cell lines, with RNU6B as internal control. Values are not comparable between MDA-MB-231 and MCF7 cells. **(C)** primary mir-29b-1 and mir-29b-2 transcript levels were detected by qRT-PCR using specific primers. 18S rRNA was used as internal control. **(D)** total cell lysates were subjected to κB-site-DNA-bait ELISA assay to detect total active NF-κB dimer level. Values are not comparable between MDA-MB-231 and MCF7 cells. **(E)** cells transfected with NF-κB reporter plasmids were harvested after 48 h, luciferase activity was analyzed in cell lysates. **(F)** activated p-p65 subunit of NF-κB in different cells were observed with confocal microscopy. Nuclei were stained with DAPI. **(G)** nuclear and cytoplasmic portions of cells were extracted and activated p65 NF-κB were detected with κB-site-DNA-bait ELISA. Data show the relative percentage of active NF-κB in the two portions. (*p < 0.05, **p < 0.01).

In order to analyze the mechanism by which S100A7 differentially regulates cell proliferation, we characterized the gene expression signatures in the microarray data of S100A7 overexpressing MDA-MB-231 and MCF7 cells (231-S100A7 and MCF7-S100A7). We postulated that we would pinpoint the driving factor of proliferative regulation by comparing the differential imprints of S100A7 overexpression in the transcriptomes of MDA-MB-231 and MCF7 cells. To do this, we performed the Gene Functional Enrichment (GFE) analysis to the microarray data of 231-V/231-S100A7 and MCF7-V/MCF7-S100A7 cells with the bioinformatic engine, *ToppGene*. We used the genes which were up-/down-regulated greater than 2 folds in 231-S100A7 (*v.s.* vector control) and a comparable number of genes which were up-/down-regulated greater than 2.5 folds in MCF7-S100A7 (*v.s.* vector control) as training sets for randomized separate GFE analysis. We then compared the GFE analysis outcomes of these four sets and discovered a common imprint between MDA-MB-231 and MCF7 that is associated with opposite changes of genes: the expression changes of miR-29b targets. As shown in Figure 1A, miR-29b target genes were significantly enriched in the genes upregulated in 231-S100A7 and also in those downregulated in MCF7-S100A7 cells. miR-29b has been considered a strong tumor suppressor in multiple cancers. Based on the fact that miR-29b target genes were upregulated in 231-S100A7 and downregulated in MCF7-S100A7 cells, we hypothesized that S100A7 differentially regulates miR-29b expression which subsequently affects cell proliferation in MDA-MB-231 and MCF7 cells through modulation of miR-29b target genes. In order to verify this, we first analyzed the expression levels of both mature miR-29b and its two primary microRNAs on chromosome 7 and 1. In agreement with our GFE analysis, we observed that S100A7 overexpression significantly downregulated miR-29b expression in MDA-MB-231 cells and upregulated miR-29b in MCF7 cells, in both mature (miR-29b) and primary (pri-mir-29b-1 and pri-mir-29b-2) forms (Figure 1B, C). Importantly, in agreement with our *in vitro* cell line data (Figure 1B, C), analysis of TCGA invasive breast cancer patient data [24] showed that S100A7 overexpression in ER^+^ patients is more likely to correlate with miR-29b upregulation than ER^−^ patients; and S100A7 overexpression in ER^−^ patients is more likely to correlate with miR-29b downregulation than ER^+^ patients (Additional file 1: Figures S1, S2 and S3). These data showed that S100A7 differentially regulates miR-29b transcription in ER^−^ and ER^+^ breast cancers.

### S100A7 differentially modulates NF-κB activation in MDA-MB-231 and MCF7

Interestingly, we observed differential regulation of NF-κB activity by S100A7, which is similar to the differential regulation of miR-29b expression in MDA-MB-231 and MCF7 cells. Using both κB-site-DNA-bait ELISA and NF-κB-luciferase reporter assay, we observed an increase of NF-κB activity in MDA-MB-231 and a decrease of NF-κB activity in MCF7 after S100A7 overexpression (Figure 1D, E). κB-site-DNA-bait ELISA detected the overall activated p65 NF-κB level, and NF-κB-luciferase reporter assay detected the actual transcription driving NF-κB level. Similar to changes in NF-κB activation level, we also observed increased NF-κB nuclear translocation in MDA-MB-231 and decreased NF-κB nuclear translocation in MCF7 after S100A7 overexpression (Figure 1F, G).

### miR-29b transcription is differentially regulated by S100A7 via NF-κB in MDA-MB-231 and MCF7

**Figure 2:**
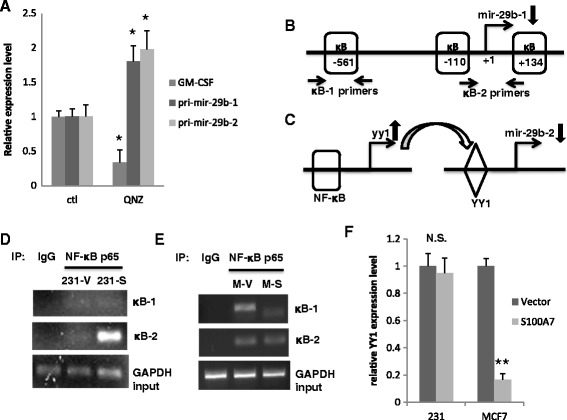
**S100A7 differentially regulates miR-29b transcription in MDA-MB-231 and MCF7 cells. (A)** MCF7 cells were treated with 5nM QNZ for 24 h before they were harvested for qRT-PCR analysis. A well-known NF-κB driven gene GM-CSF served as the positive control for effect of QNZ inhibition. **(B)** κB binding sites at the proximal of mir-29b-1 gene transcriptional start site. Two sets of primers, κB-1 and κB-2, were designed to detect three κB binding sites in ChIP. **(C)** indirect inhibition of mir-29b-2 transcription by NF-κB and YY1. **(D)** ChIP assay of NF-κB p65 subunit binding to mir-29b-1 promoter region in 231-V and 231-S cells. **(E)** ChIP assay of NF-κB p65 subunit binding to mir-29b-1 promoter region in MCF7-V (M-V) and MCF7-S (M-S). **(F)** qRT-PCR quantification of YY1 level in different cells. (*p < 0.05, **p < 0.01, N.S.: non-significant).

miR-29b expression has been shown to be inhibited by NF-κB in non-breast cancer cells [18,19,25]. We intended to find out whether S100A7 affected miR-29b transcription via regulating NF-κB activation in MDA-MB-231 and MCF7. We first verified that, in breast cancer cells, inhibiting NF-κB activation with its inhibitor, QNZ, significantly enhanced the transcription of pri-mir-29b-1 and pri-mir-29b-2 (Figure 2A). This showed that NF-κB activity was inversely correlated with miR-29b transcription in breast cancer cells. pri-mir-29b-1 promoter contains three NF-κB binding sites. The binding of NF-κB to the promoter directly suppresses pri-mir-29b-1 transcription (Figure 2B). In addition, NF-κB transactivates the transcriptional factor, YY1, which in turn can bind to the promoter of pri-mir-29b-2 to suppress its transcription (Figure 2C). By ChIP assay and qRT-PCR, we observed that S100A7 overexpression differentially altered the binding of NF-κB to the promoter of pri-mir-29b-1 and/or the expression of pri-mir-29b-2 suppressor, YY1, in MDA-MB-231 and MCF7 cells (Figure 2D-F). Both direct and indirect transcriptional regulations by NF-κB led to the differential regulation of miR-29b levels by S100A7, in MDA-MB-231 and MCF7 cells.

### S100A7 downregulates PI3K p85α and CDC42 via targeting of miR-29b to activate and stabilize p53 in MCF7

**Figure 3:**
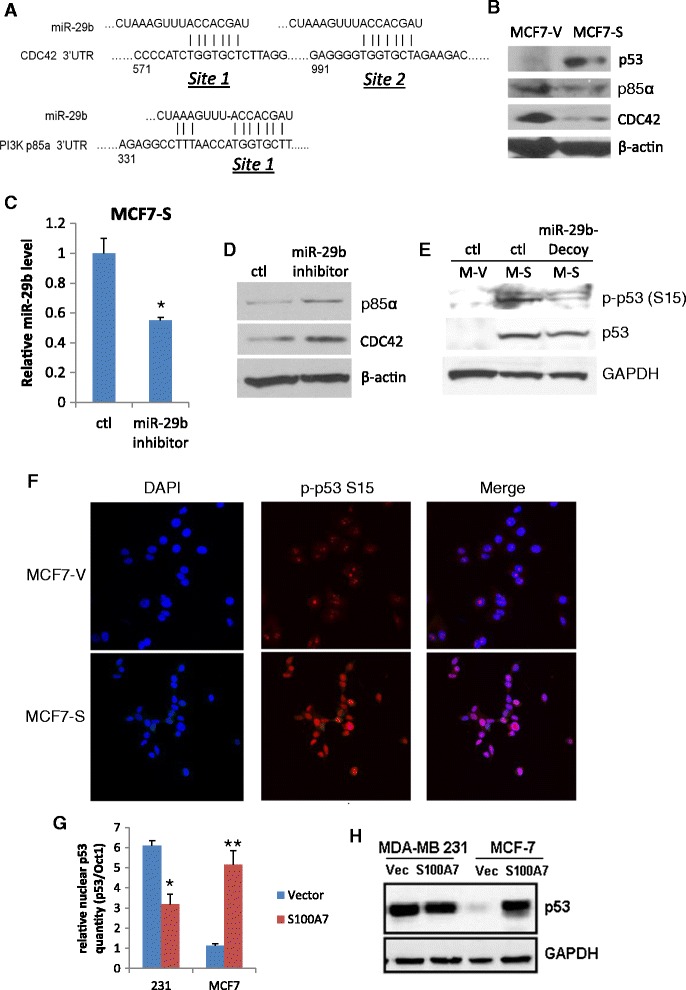
**miR-29b targets p85α and CDC42, and determines the effects of S100A7 on p53 activation and stability in different cells. (A)** target sites of miR-29b on 3′ UTRs of CDC42 and p85α mRNAs. **(B)** WB of p53, p85α and CDC42. β-actin is loading control. **(C)** miR-29b knockdown by antagomir inhibitor in MCF7-S cells. **(D)** WB of transient miR-29b inhibitor transfection partially rescuing p85α and CDC42 protein expression in MCF7-S cells. **(E)** prolonged knock down of miR-29b with miR-29b-Decoy reduced p53 phosphorylation and total p53 level in MCF7-S cells. **(F)** Confocal microscopy of phosphorylated p53 localization in MCF7-V and MCF7-S cells. Nuclei were labelled with DAPI. **(G)** relative p53 levels in nuclear extract of cells. p53 levels were normalized to nuclear marker Oct1. **(H)** total p53 levels of cells indicating the stability of p53. (*p < 0.05, **p < 0.01).

It has been reported that miR-29b activates p53 through targeting the 3′ UTR of the oncogenes, PI3K p85α and CDC42 [22] (Figure 3A). Activated (phosphorylated) p53 translocates into nucleus and is protected from degradation in the cytoplasm (stabilized). We showed that S100A7 induced upregulation of miR-29b in MCF7 cells was able to reduce the level of p85α and CDC42 proteins (Figure 3B). Transient knockdown of miR-29b with miR-29b inhibitor (antagomir) partially rescued the expression of p85α and CDC42 (Figure 3C, D). Prolonged knock down of miR-29b by miR-29b-Decoy transfection reduced p53 activation in MCF7-S100A7 (Figure 3E). Compared to MDA-MB-231, S100A7 caused a much more dramatic change of miR-29b in MCF7 cells (Figure 1B). This dramatic rise of miR-29b led to a significant increase of p53 phosphorylation, nuclear translocation (Figure 3E-G, Additional file 1: Figure S4) and total p53 protein level (Figure 3H) in MCF7-S100A7 cells.

### S100A7 enhances p53 related anti-proliferative pathways in MCF7

**Figure 4:**
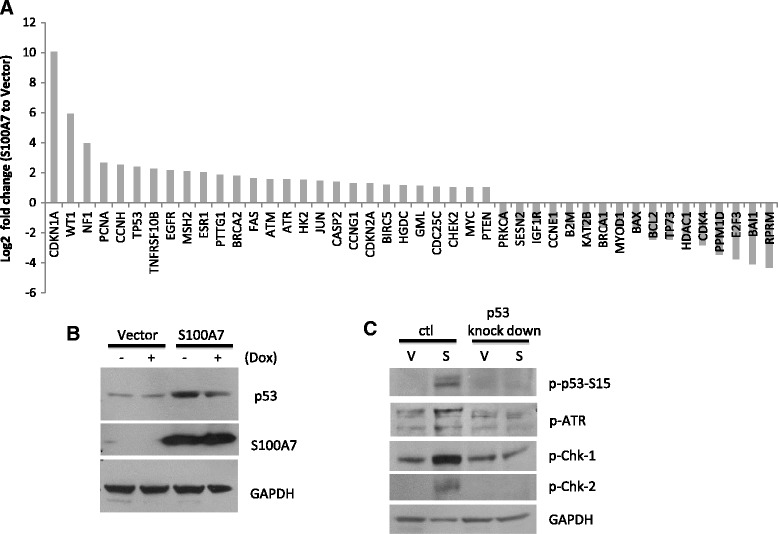
**S100A7 activated p53 related anti-proliferative pathways. (A)** Log_2_ fold change of mRNA expression of p53 pathway factors after S100A7 overexpression. **(B)** WB of p53 and S100A7 in MCF7-p53-CKD transfected with S100A7 or vector control. p53 shRNA transcription and p53 knockdown in MCF7-p53-CKD was induced by Doxycyclin (Dox). **(C)** S100A7 activated p53, ATR, chk-1, chk-2 in MCF7 cells.

We further investigated the mechanism by which S100A7 inhibited MCF7 proliferation. p53 pathway qRT-PCR array data revealed that S100A7 overexpression in MCF7 cells led to the upregulation of p53 activating factors (such as ATM, ATR, CDKN1A/p21, CDKN2A and CHEK2), p53 targeted apoptotic factors (such as FAS and TNFRSF10B), p53 targeted DNA repairing factors (such as PCNA and PTTG1) and p53 targeted anti-survival factors (such as PTEN) (Figure 4A). Consequently, DNA repair pathway in MCF7-S100A7 cells was also activated through p53. Phosphorylation of ATR, Chk-1 and Chk-2 was enhanced by S100A7, which was absent when p53 is knocked down in MCF7 cells [26] (Figure 4B, C). S100A7 induced p53 upregulation appeared to be concurrently related with significantly enhanced anti-proliferative pathways in MCF7 cells.

### S100A7 induced differential regulation of miR-29b is important for its differential effects on cell proliferation of MDA-MB-231 and MCF7 *in vitro* and their tumor growth *in vivo*

**Figure 5:**
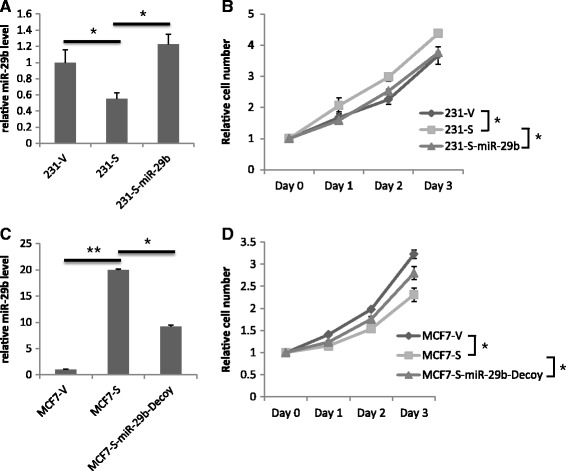
**Manipulating miR-29b could counteract the effects of S100A7 on cell proliferation in MDA-MB-231 and MCF7.** The proliferation of MDA-MB-231 and MCF7 cell lines were determined by MTT assay. **(A, C)** miR-29b levels of indicated cell lines. **(B, D)** MTT cell proliferation assays of indicated cell lines. (*p < 0.05, **p < 0.01).

**Figure 6:**
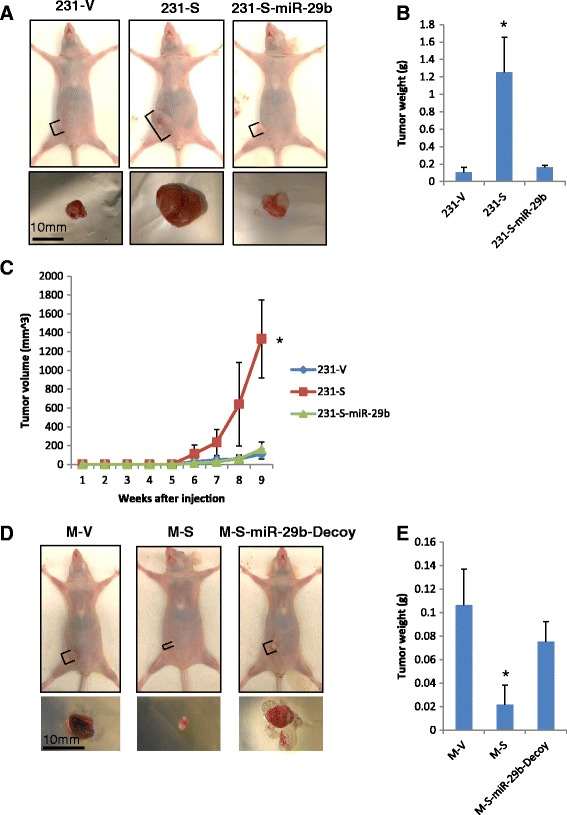
**miR-29b determines the effects of S100A7 on tumor growth of MDA-MB-231 and MCF7 cells. (A and D)** representing tumors of each group at the end of study. Brackets mark the size of tumors on mice. All tumors are to the same scale. **(B and E)** tumor weight of each group. *p < 0.05 as analyzed by ANOVA. 231-V and 231-S-miR-29b are not significantly different, neither are MCF7-V and MCF7-S-miR-29b-Decoy. **(C)** tumor volume of each group. *p < 0.05 as analyzed by ANOVA. 231-V and 231-S-miR-29b are not significantly different.

To verify the importance of miR-29b in determining the differential effects of S100A7, we stably transfected 231-S100A7 and MCF7-S100A7 cells with exogenous miR-29b and miR-29b-Decoy, respectively, to reverse the changes of miR-29b caused by S100A7 (Figure 5A, C). The S100A7 induced proliferation increase in MDA-MB-231 was repressed by exogenous miR-29b (Figure 5B), and the decrease of proliferation in MCF7 was also partially rescued by miR-29b-Decoy (Figure 5D, Additional file 1: Figure S5). When we orthotopically injected these cells into nude mice mammary fat pad, miR-29b overexpression repressed S100A7 induced tumor growth of MDA-MB-231 cells (Figure 6A-C), whereas miR-29b knockdown rescued tumor growth from S100A7 suppression in MCF7 cells (Figure 6D, E). Thus, we confirmed that miR-29b is the determining factor of the differential effects of S100A7 in ER^−^ and ER^+^ breast cancer cells.

## Discussion

**Figure 7:**
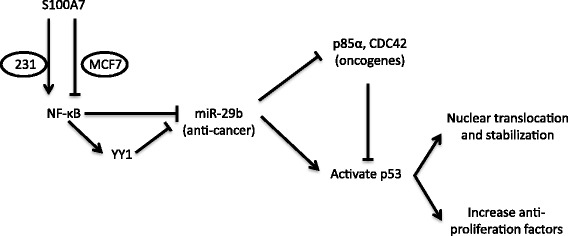
**Proposed mechanism of differential regulations of NF-κB – miR-29b – p53 pathway by S100A7 in different types of breast cancer cells.**

In the present article, we proposed a novel mechanism for the differential roles of the inflammatory protein S100A7 on the proliferation of ER^−^ and ER^+^ breast cancer cells. S100A7 can either promote or suppress breast cancer cell proliferation through distinct modulation of the NF-κB – miR-29b – p53 pathway in ER^−^ MDA-MB-231 and ER^+^ MCF7 cells, respectively (summarized in Figure 7).

miR-29b governs numerous genes’ expression by targeting their 3′ UTRs, leading to both translational suppression and instability of mRNAs. Since large portions of these regulated genes are associated with cell proliferation, miR-29b has been considered as a tumor suppressor in various cancers [14,15,19,27], including breast cancer [16,17]. Moreover, other than regulation of cell proliferation, miR-29b has also been shown to inhibit breast cancer metastasis [16]. Through bioinformatic analysis and *in vitro* assays, we found that miR-29b expression was reduced by S100A7 in ER^−^ MDA-MB-231 and increased by S100A7 in ER^+^ MCF7 cells, which at least partly explained the different roles of S100A7 in regulating proliferation of different types of breast cancer cells. The clinical relevance of this was supported by patient data of the TCGA cohort, which showed that S100A7 upregulation is more likely to be associated with increased miR-29b expression in ER^+^ breast cancer patients than ER^−^ patients and *vice versa*. Interestingly, we noticed that, although S100A7 is overexpressed in both ER^−^ and ER^+^ breast cancer patients, there is a significant mutual-exclusivity of S100A7 and ER expression in human breast tumors (Additional file 1: Figures S1–S2). In other words, S100A7 overexpression is more commonly seen in ER^−^ tumors. This is in agreement with previous works based on different cohorts of breast cancer patients [5].

Moreover, the differential regulation of miR-29b expression was associated with differential regulation of NF-κB activation by S100A7. NF-κB has been shown to either directly or indirectly inhibit the expression of miR-29b, which is transcribed from mir-29b-1 on chromosome 7 and mir-29b-2 on chromosome 1 [18,19]. Directly, NF-κB can bind to the promoter of mir-29b-1 to block its transcription. Indirectly, NF-κB can transactivate YY1 which then block the transcription of mir-29b-2 by binding to its promoter [19]. In the present study, we showed that S100A7 enhanced NF-κB activity in MDA-MB-231 and inhibited NF-κB activity in MCF7, which then directly and/or indirectly influences miR-29b transcription. It was known that ER negatively regulates activity in breast cancer [21,28-30], and may even block the transcriptional function of NF-κB [30]. Thus, the absence/presence of ER in breast cancer cells may lead to the differential regulations of NF-κB activation by S100A7 in MDA-MB-231 (ER^−^) and MCF7 (ER^+^) cells. In this work, ER status was used as the primary standard for dividing breast cancer cells into two groups with distinct responses to S100A7. To our knowledge, the distinct regulation of NF- κB - miR-29b by S100A7 is shown for the first time.

Among the targets of miR-29b, PI3K p85α and CDC42 have been shown to be closely related to cancer growth [22,31,32]. And, as oncogenes, they inhibit the activation of tumor suppressor, p53 [22]. Activated p53 translocates into the nucleus and avoids degradation in the cytoplasm. Using MCF7, whose miR-29b was more significantly affected by S100A7, we showed that miR-29b targeted PI3K p85α and CDC42, which consequently increased p53 level by enhancing its activation and nuclear translocation.

Activated p53 can enhance multiple anti-proliferative pathways to inhibit cancer growth [23]. Here, we showed that increased p53 activation and total protein level increased the expression of multiple p53 downstream anti-proliferative factors, including mitosis checkpoint pathways and DNA repair pathways. These effects were considered to exert anti-proliferative influences to breast cancer cells.

In addition, we reversed the effects of S100A7 on cell proliferation and tumor growth by overexpressing miR-29b in 231-S100A7 cells and knocking down miR-29b in MCF7-S100A7 cells, which reflected that miR-29b is not only sufficient but also necessary for determining the differential effects of S100A7 in breast cancer cells. This demonstrated that miR-29b functions downstream of S100A7 and is important in determining the differential effects of S100A7 in breast cancer cells.

In conclusion, we report a novel regulatory route that determines the pro-/anti-proliferative roles of S100A7 in ER^−^ and ER^+^ breast cancer. In these different types of breast cancer cells, S100A7 differentially regulates NF-κB activation, which then differentially affects miR-29b expression and p53 functions. And reversing miR-29b changes can suppress the effects of S100A7 in these cells. Thus, it is suggested that miR-29b may be paired with S100A7 to serve as more accurate biomarkers for breast cancer diagnosis and prognosis. On the other hand, S100A7 does not have kinase activity, which makes it difficult to be targeted with drugs. Recently, many lipid-nanoparticle-based technologies have made it possible to target miRNAs in clinical trials of cancer treatment [33]. Hence, with the discovery of S100A7 - miR-29b regulatory route, miR-29b may be potential to serve as an alternative target for S100A7 overexpression in breast cancer treatment.

## Materials and methods

### Reagents and cells

NF-κB p65, p53, PI3K p85α, CDC42, Oct1, β-actin and GAPDH antibodies and NF-κB inhibitor QNZ were purchased from Santa Cruz Biotechnology (Santa Cruz, CA). miR-29b real-time PCR assay kit and miR-29b inhibitor were purchased from Life Technologies (Carlsbad, CA). Luciferase substrate was purchased from Promega (Madison, WI). p-p53 (S15), p-ATR, p-Chk-1, p-Chk-2 antibodies were purchased from Cell Signaling Techology (Danvers, MA). S100A7 antibody was purchased from Novus Biologicals (Littleton, CO). MDA-MB-231 cell and its derivatives were cultured in DMEM medium supplemented with 10% FBS and 1% dual antibiotics. MCF7 cell and their derivatives were cultured in MEMα medium supplemented with 10% FBS and 1% dual antibiotics. p53-conditional-knockdown-MCF7 cell line was provided by Dr. Jill Bargonetti (The City University of New York). 231-S-miR-29b cell was generated by stably transfecting pcDNA3-miR29b plasmid shared by Dr. Joshua Mendell (Johns Hopkins University) [34]. MCF7-S-miR-29b-Decoy cell was generated by stably transfecting AB.pCCL.sin.cPPT.U6.miR-29b-Decoy.hPGK.GFP.WPRE plasmid shared by Dr. Brian Brown (Mount Sinai School of Medicine) [35].

### Animal study

Adult female nude mice (NU/NU), obtained from Charles River Laboratories (Wilmington, MA), were housed under specific pathogen-free conditions. MDA-MB-231, MCF7 or their derivatives (3×10^6^ cells/200 μL PBS) were injected into the right mammary fat pad of each mouse. Mice with MCF7 cell or derivatives were also injected subcutaneously with 2.5 μg of β-estradiol 17-valerate in 50 μL of Sesame oil once a week. Tumor size was assessed once a week and tumor volume was calculated using digital calipers according to the formula: volume = length × (width)^2^ / 2. Mice were sacrificed at the end of study or per the request of the veterinarian. All mice were kept in OSU’s animal facility in compliance with the guidelines and protocols approved by the IACUC.

### Gene functional enrichment analysis

Gene functional enrichment (GFE) analysis is performed using the bioinformatics engine, ToppGene [36], developed by the Division of Biomedical Informatics, Cincinnati Children’s Hospital Medical Center (Cincinnati, OH). Training gene list was determined by gating the significantly up-/down-regulated genes from microarray data as specified. Enrichment analysis was corrected by Bonferroni method with a p-Value cutoff of 0.05.

### Quantitative reverse transcription–PCR (qRT-PCR)

qRT-PCR was performed as previously described [37]. Briefly, total RNA was extracted from cells using TRIzol reagent (Life Technologies) and purified with RNeasy kit (Qiagen, Valencia, CA). For mRNA detection, total RNA was then reverse transcribed into cDNA using High Capacity cDNA Reverse Transcription Kit (Life Technologies) and quantified using Power SYBR Green Master Mix (Life Technologies). p53 pathway PCR array was performed using p53 Signaling Pathway RT2 Profiler PCR Array from Qiagen. For miRNA detection, total RNA was reverse transcribed and miRNAs quantified using miRNA specific real-time PCR kits from Life Technologies. Real-time PCR was performed on Eppendorf Mastercycler realplex. Data analysis was performed using standard “delta delta Ct method”.

### NF-κB activity assays

For the κB-site-DNA-bait ELISA assay, nuclear and cytoplasmic extractions of cells were prepared using NE-PER Nuclear and Cytoplasmic Extraction Reagents (Thermo Scientific) per the product manual. Activated NF-κB p65 levels of both nuclear and cytoplasmic extracts were measured using TransAM NF-κB p65 Transcription Factor ELISA Kit (Active Motif, Carlsbad, CA) per the product manual. For the luciferase reporter assay, cells were transfected with equal amount of pNF-κB-Luc reporter plasmids by lipofectamine 2000 (Life Technologies) and harvested after 48 h. Cells were lysed and luciferase activity was measured with the luciferase assay kit from Promega (Madison, WI) and Synergy plate reader (BioTek, Winooski, VT) per the manufacturer’s manual.

### Confocal microscopy

Cells grown on micro-chamber-slides were fixed and permeabilized using the Fix/Perm solution (BD Biosciences, San Jose, CA) for 20 min at 4°C. The cells were washed with BD Perm/Wash buffer (BD Biosciences), and stained with respective primary antibodies for 1 h at 4°C. After the incubation, cells were washed and stained with Alexa Fluor 488 or 568 secondary antibodies for 1 h at 4°C. The slides were mounted using Vectashield mounting medium with DAPI (Vector Laboratories) and then imaged with Olympus FV1000 confocal microscope. The pictures were processed using Olympus FV-10 ASW software.

### Chromatin immunoprecipitation (ChIP)

Cells were fixed and cross-linked in 2% paraformaldehyde, and then cross-linking was quenched with 10% volume of 1.375 M glycine. After washing with ice cold PBS, cells were harvested and lysed with NP-40 ChIP buffer. Nuclei were isolated by centrifuge, and lysed with SDS nuclei lysis buffer. DNA was digested using Micrococcal Nuclease from New England Biolabs, and digestion was stopped by adding 50 mM EDTA. DNA samples were then immunoprecipitated overnight at 4°C with NF-κB p65 antibody and Protein G/Protein A Agarose Beads (Calbiochem). After washing, DNA was eluted using SDS elution buffer, and then reverse cross-linked by incubation overnight at 65°C. DNA was purified using TRIzol reagent (Life Technologies) and chloroform, and then subjected to PCR with specific primers.

### Cell proliferation assay

Cell proliferation assay was performed using Cell Proliferation Kit I (MTT) from Roche (Indianapolis, IN) per the kit manual. Proliferation assays were done in triplicates. O.D. values were measured using Model 680 microplate reader from BIO-RAD (Hercules, CA).

### Western blotting (WB)

WB was performed as previously described [37]. Briefly, proteins in cell lysates were separated by electrophoresis using NuPAGE SDS PAGE Gel (Life Technologies). Proteins transferred onto Nitrocellulose membrane were then blotted by specific primary and HRP-conjugated secondary antibodies. Protein expression was detected by Thermo ECL reagents using X-ray films.

### Statistical analysis

Reported data for cell line studies are the means ± S.E.M. of at least three independent experiments performed in duplicates or triplicates. Animal studies use n = 5 ~ 8 mice for each group. The statistical significance was determined by the Student’s t test or as specified.

## Additional file

Additional file 1:
**Supplementary figures, legends and references.**
