# Erratum to: miR-29b defines the pro-/anti-proliferative effects of S100A7 in breast cancer

**DOI:** 10.1186/s12943-015-0451-9

**Published:** 2015-11-16

**Authors:** Helong Zhao, Tasha Wilkie, Yadwinder Deol, Amita Sneh, Akaansha Ganju, Mustafa Basree, Mohd W. Nasser, Ramesh K. Ganju

**Affiliations:** Department of Pathology, The Ohio State University Wexner Medical Center, 840 BRT, 460 W 12th Ave, Columbus, 43210 OH USA

Erratum

After publication of [1] it came to the authors’ attention that in the abstract. The sentence “In the present study, we show that S100A7 significantly downregulates the expression of miR-29b in Estrogen Receptor (ER)-positive breast cancer cells (represented by MCF7), and significantly upregulates miR-29b in ER-negative cells (represented by MDA-MB-231)”. Was Incorrect, the correct statement is “In the present study, we show that S100A7 significantly upregulates the expression of miR-29b in Estrogen Receptor (ER)-positive breast cancer cells (represented by MCF7), and significantly downregulates miR-29b in ER-negative cells (represented by MDA-MB-231)”.
