# Supplementary material for: miR-29b defines the pro-/anti-proliferative effects of S100A7 in breast cancer
Source: Mol Cancer. 2015 Jan 27;14:11. doi: 10.1186/s12943-014-0275-z (PMC4314775; doi:10.1186/s12943-014-0275-z)
Supplement: Additional file 1: — Supplementary figures, legends and references. [file 12943_2014_275_MOESM1_ESM.pdf]

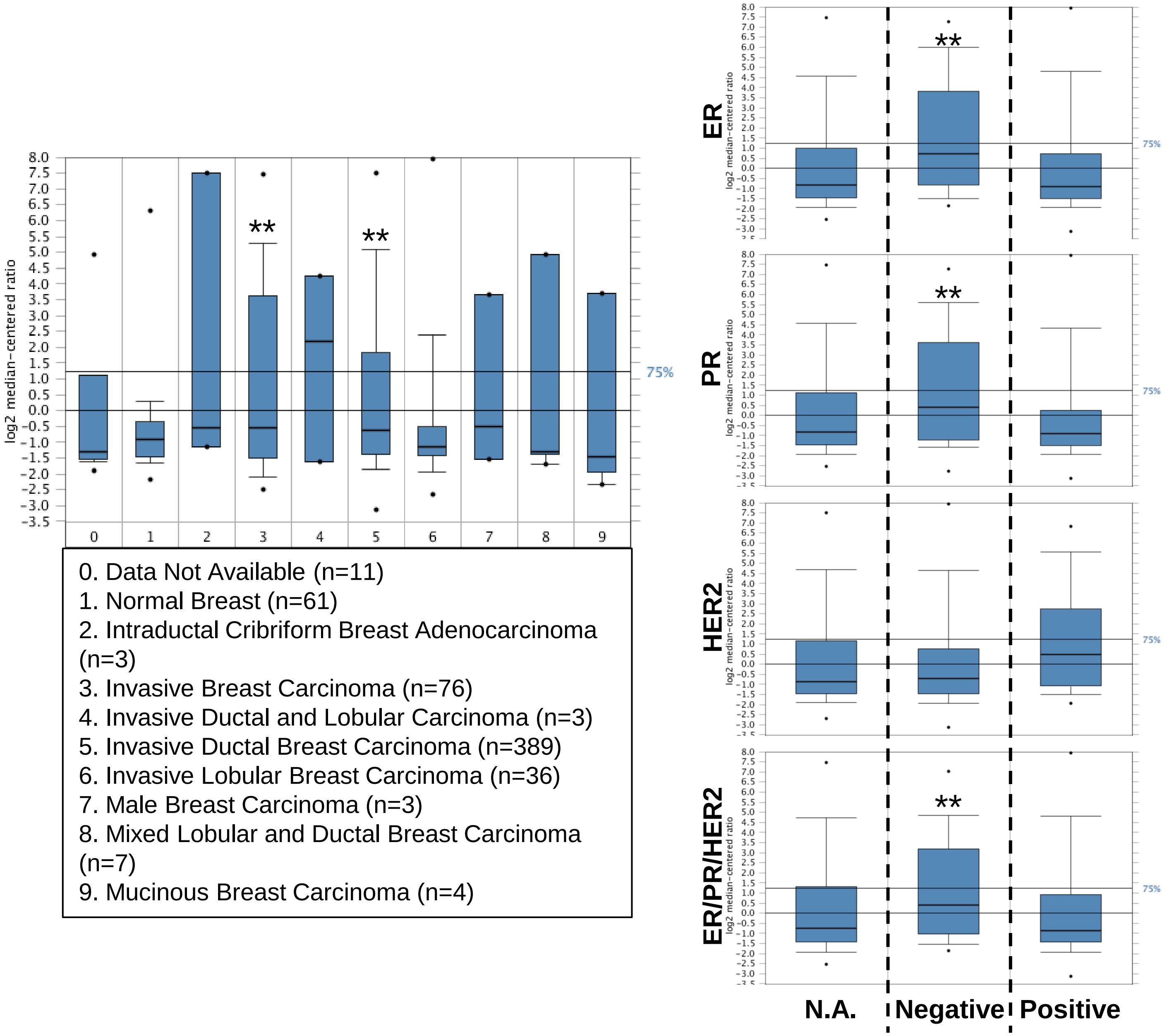

Figure S1. Expression of S100A7 in different subtypes of invasive breast cancer patients of the TCGA database. mRNA expression levels of S100A7 in different subtypes were analyzed among the 825 available patient samples in TCGA[2] invasive breast cancer database. (Left Panel) Patients were grouped by cancer type. **p<0.01 comparing to normal breast tissue. (Right Panel) Patients were grouped by receptor status. **p<0.01 comparing to ungrouped (data not available/N.A.) and positive status. 75 percentile of S100A7 upregulation is labeled in each figure. Figures were generated through Oncomine[1].

Gene	S100A7 ↑	ER$^-$	miR-29b ↑	miR-29b ↓
S100A7 ↑		$p<10^{-6}$	$p=7.6\times10^{-5}$	$p=0.059$
ER$^-$			$p=5.5\times10^{-3}$	$p=2.1\times10^{-4}$
miR-29b ↑				$p=0.00$
miR-29b ↓				

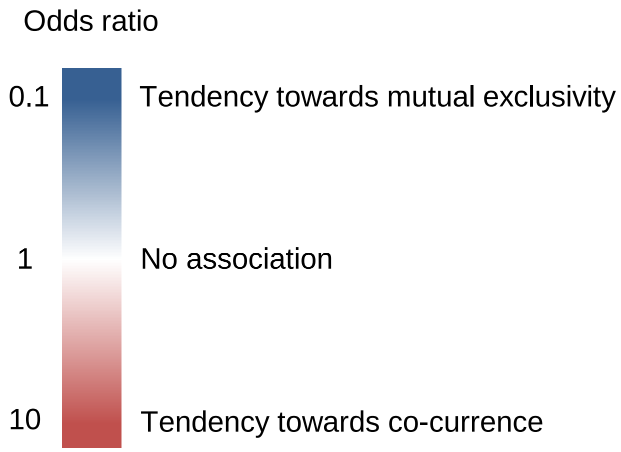

Condition	Definition
S100A7 ↑	S100A7: EXPRESSION > MEAN - 0.1×SD
ESR1$^-$	ESR1: EXPRESSION < MEAN + 0.1×SD
miR-29b ↑	MIR-29B-1/-2/29B: EXPRESSION > MEAN + 0×SD
miR-29b ↓	MIR-29B-1/-2/29B: EXPRESSION < MEAN + 0×SD

Figure S2. Mutual exclusive analysis of S100A7 overexpression, ER status and miR-29b up-/down-regulation in invasive breast cancer patients of the TCGA database. mRNA/miRNA expression levels of S100A7, ESR1, MIR-29-1/29B and MIR-29B-2/29B were analyzed among the 825 available patient samples in TCGA[2] invasive breast cancer database. In TCGA cohort, most patients have ER-positive tumors, with only 22.95% patients have ER-negative tumors[1]. To match this, ER-negative is defined as ESR1 mRNA expression being less than 0.1SD above the mean value of the whole cohort, the rest being ER-positive In invasive breast cancer patients, 26.32% of them have S100A7 overexpression[3]. To match this, S100A7 overexpression is defined as S100A7 mRNA expression being greater than 0.1SD below the mean. miR-29b upregulation is defined as MIR-29B-1/29B and MIR-29B-2/29B expression being greater than the mean, and downregulation is being less than the mean. p values of Fisher's Exact Test are shown in the table. Color coding indicates the odds ratio.
S100A7 upregulation is enriched in ER-negative cancers, and is simultaneously associate with miR-29b downregulation.

	Number of patients (Red=TRUE, White=FALSE)							
•S100A7 overexpression	215				610			
•ER-negative	126		89		83		527	
•MIR-29B-1 upregulation	30	96	27	62	19	64	67	460
•MIR-29B-2 upregulation	30	96	27	62	19	64	67	460
	% of patients (Red=TRUE, white=FALSE)							
•S100A7 overexpression	26.06%				73.94%			
•ER-negative	58.60%		41.40%		13.61%		86.39%	
•MIR-29B-1 upregulation	23.81%	76.19%	30.34%	69.66%	22.89%	77.11%	12.71%	87.29%
•MIR-29B-2 upregulation	23.81%	76.19%	30.34%	69.66%	22.89%	77.11%	12.71%	87.29%

	Number of patients (Red=TRUE, white=FALSE)							
•S100A7 overexpression	215				610			
•ER-negative	126		89		83		527	
•MIR-29B-1 downregulation	28	98	17	72	25	58	70	457
•MIR-29B-2 downregulation	28	98	17	72	25	58	70	457
	% of patients (Red=TRUE, white=FALSE)							
•S100A7 overexpression	26.06%				73.94%			
•ER-negative	58.60%		41.40%		13.61%		86.39%	
•MIR-29B-1 downregulation	22.22%	77.78%	19.10%	80.90%	30.12%	69.88%	13.28%	86.72%
•MIR-29B-2 downregulation	22.22%	77.78%	19.10%	80.90%	30.12%	69.88%	13.28%	86.72%

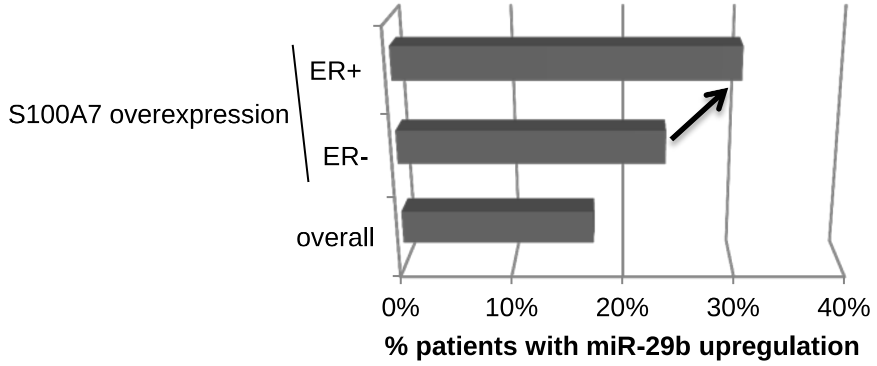

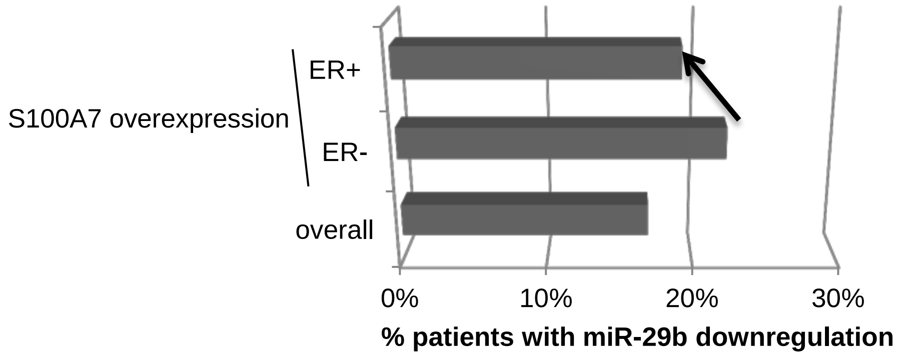

Figure S3

Figure S3. Influence of S100A7 overexpression on miR-29b expression level in invasive breast cancer patients of the TCGA database. mRNA/miRNA expression levels of S100A7, ESR1, MIR-29-1/29B and MIR-29B-2/29B were analyzed among the 825 available patient samples in TCGA[2] invasive breast cancer database. Definitions of conditions are same as Figure S1. Number of patients and its percentage is shown in each cell of table. Data are summarized in bar graphs. S100A7 overexpression in ER^+ patients is more likely to correlate with miR-29b upregulation than ER^- patients. Meanwhile, S100A7 overexpression in ER^- patients is more likely to correlate with miR-29b downregulation than ER^+ patients.

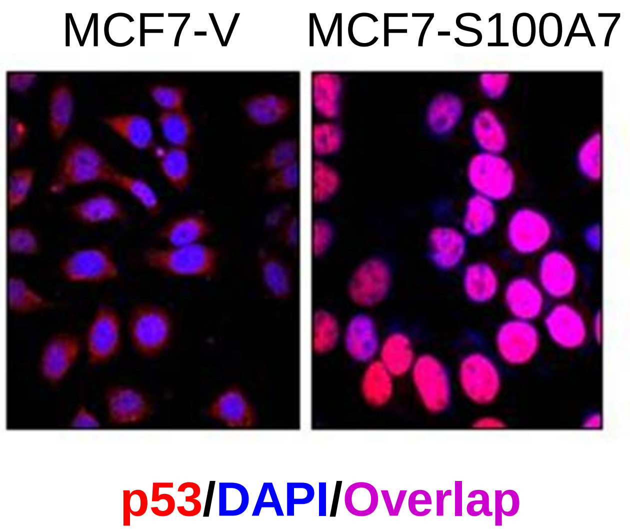

Figure S4. p53 localization in MCF7-V and MCF7-S100A7 cells. p53 aggregated in the nucleus of MCF7-S100A7 cells, while it mainly resided in the cytoplasm of MCF7-V cells. MCF7-V and MCF7-S100A7 cells were fixed, permeablized and stained for p53 and nucleus (DAPI). Confocal images are the merge of two colors.

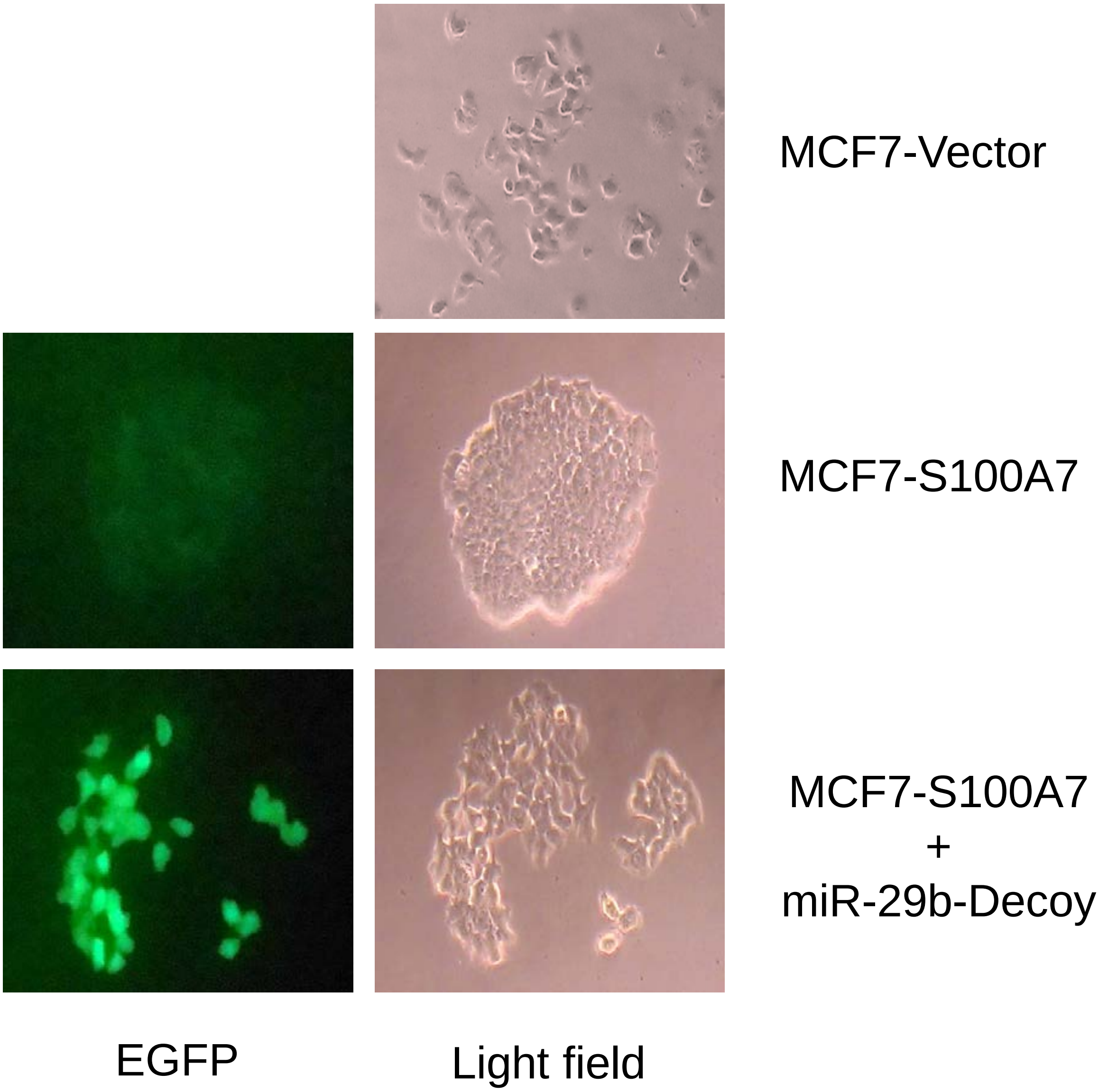

Figure S5. miR-29b-Decoy transfection reversed the phenotypic changes (epithelial transition) induced by S100A7 in MCF7 cells. 48h after miR-29b-Decoy transfection in MCF7-S100A7, its morphology was examined under microscope. EGFP expression indicates cells transfected with miR-29b-Decoy plasmids.

Supplementary references:

1. Rhodes DR, Kalyana-Sundaram S, Mahavisno V, *et al* (2007). Oncomine 3.0: Genes, Pathways, and Networks in a Collection of 18,000 Cancer Gene Expression Profiles. *Neoplasia* **9:**166-180.

2. Al-Haddad S, Zhang Z, Leygue E, Snell L, Huang A, Niu Y *et al* (1999). Psoriasin (S100A7) expression and invasive breast cancer. *Am J Pathol* **155:** 2057-2066.

3. Network CGA (2012). Comprehensive molecular portraits of human breast tumours. *Nature* **490:** 61-70.
